# Functional analysis of epilepsy‐associated GABA_A_ receptor mutations using *Caenorhabditis elegans*

**DOI:** 10.1002/epi4.12982

**Published:** 2024-05-30

**Authors:** Ami Gadhia, Eleanor Barker, Alan Morgan, Jeff W. Barclay

**Affiliations:** ^1^ Department of Biochemistry, Cell and Systems Biology, ISMIB University of Liverpool Liverpool UK

**Keywords:** convulsion, locomotion, seizure, shrinker, unc‐49

## Abstract

**Objective:**

GABA_A_ receptor subunit mutations pose a significant risk for genetic generalized epilepsy; however, there are over 150 identified variants, many with unknown or unvalidated pathogenicity. We aimed to develop in vivo models for testing GABA_A_ receptor variants using the model organism, *Caenorhabditis elegans*.

**Methods:**

CRISPR‐Cas9 gene editing was used to create a complete deletion of *unc‐49*, a *C. elegans* GABA_A_ receptor, and to create homozygous epilepsy‐associated mutations in the endogenous *unc‐49* gene. The *unc‐49* deletion strain was rescued with transgenes for either the *C. elegans unc‐49B* subunit or the α1, β3, and γ2 subunits for the human GABA_A_ receptor. All newly created strains were analyzed for phenotype and compared against existing *unc‐49* mutations.

**Results:**

Nematodes with a full genetic deletion of the entire *unc‐49* locus were compared with existing *unc‐49* mutations in three separate phenotypic assays—coordinated locomotion, shrinker frequency and seizure‐like convulsions. The full *unc‐49* deletion exhibited reduced locomotion and increased shrinker frequency and PTZ‐induced convulsions, but were not found to be phenotypically stronger than existing *unc‐49* mutations. Rescue with the *unc‐49B* subunit or creation of humanized worms for the GABA_A_ receptor both showed partial phenotypic rescue for all three phenotypes investigated. Finally, two epilepsy‐associated variants were analyzed and deemed to be *loss of function*, thus validating their pathogenicity.

**Significance:**

These findings establish *C. elegans* as a genetic model to investigate GABA_A_ receptor mutations and delineate a platform for validating associated variants in any epilepsy‐associated gene.

**Plain Language Summary:**

Epilepsy is a complex human disease that can be caused by mutations in specific genes. Many possible mutations have been identified, but it is unknown for most of them whether they cause the disease. We tested the role of mutations in one specific gene using a small microscopic worm as an animal model. Our results establish this worm as a model for epilepsy and confirm that the two unknown mutations are likely to cause the disease.

**Key points**

- GABA_A_ receptor mutations are associated with genetic generalized epilepsy.
- Genetic deletion of the GABA_A_ worm orthologue, *unc‐49*, exhibits functional defects including an increase in seizure‐like convulsions.
- The human GABA_A_ receptor can partially functionally replace *unc‐49*.
- Epilepsy‐associated variants of GABA_A_ have functional defects including an increase in seizure‐like convulsions.
- *C. elegans* are an inexpensive in vivo model to investigate GABA_A_ receptor mutations and epilepsy in general.

## 1 INTRODUCTION

Epilepsy is a heterogeneous group of neurological disorders primarily characterized by the presence of recurrent seizures.^1^ Although the disease can arise spontaneously, it is estimated that 30%–40% of total cases are genetic in basis. The continued and expanding use of genetic tools such as genome‐wide association studies of epilepsy patients has resulted in an increasing number of genes and mutations implicated in contributing to or causing various forms of genetic epilepsy.^2^ Identification of potential epilepsy mutations, however, is not sufficient in order to understand fully the underlying disease mechanisms and tailor individual treatment for specific genotypes. The use of varied non‐mammalian organisms has become increasingly important in epilepsy research.^3^, ^4^, ^5^, ^6^ The nematode, *Caenorhabditis elegans*, is one such genetically amenable whole‐animal model used for confirming pathogenicity of putative epilepsy mutations, investigating underlying mechanisms and screening for anti‐seizure drugs.^7^, ^8^, ^9^, ^10^

γ‐aminobutyric acid (GABA) is the main inhibitory neurotransmitter in the brain, where it signals through two major classes of receptors, the cys‐loop ligand‐gated ion channel GABA_A_ receptors, and the G‐protein‐coupled GABA_B_ receptors. Additionally, GABA_C_ receptors are a subclass of ion channels that are insensitive to typical allosteric modulators of GABA receptors.^11^ GABA signaling has long been associated with epilepsy and the onset of seizures.^12^ Indeed, GABA_A_ receptor antagonists such as pentylenetetrazole (PTZ) are known to produce seizures, whereas receptor agonists are suppressive. Within that context, it is unsurprising that a recent exome‐based case control study identified functionally relevant variants in genes encoding GABA_A_ receptor subunits as a significant risk for genetic generalized epilepsy.^13^ Indeed, there are currently over 150 epilepsy‐associated variants in the major GABA_A_ receptor subunits associated with a wide variety of symptoms and severity^14^, ^15^; however, a complete understanding of the effects of individual variants is lacking.

In the *C. elegans* genome, there are seven known ionotropic GABA receptors^16^ including the GABA_A_ homologue *unc‐49*.^17^ In contrast to vertebrate GABA_A_ receptors that are assembled from three different gene subunits, *C. elegans* requires only *unc‐49*, which can produce three distinct subunits (UNC‐49A, UNC‐49B, and UNC‐49C) through alternative splicing.^18^ Of the three isoforms, existing evidence suggests that UNC‐49A expression is barely detectable. UNC‐49B and UNC‐49C, however, are highly expressed and can form both homomeric and heteromeric functional receptors.^19^ In worms, *unc‐49* is mainly expressed postsynaptically and at body‐wall neuromuscular junctions, where it plays an inhibitory role. Loss‐of‐function alleles are characterized by uncoordinated locomotion, a shrinker phenotype, and hypersensitivity to PTZ‐induced convulsions.^3^, ^8^, ^20^, ^21^ Existing *unc‐49* mutations can affect individual or all isoforms; however, there are currently no clean deletions available for the entire gene locus.

To investigate the role of GABA_A_ receptors in epilepsy, we have created a complete genetic deletion of the *unc‐49* locus in *C. elegans* by CRISPR‐Cas9 gene editing and characterized it against existing *unc‐49* mutant alleles. To determine whether *unc‐49* can be replaced functionally by human GABA_A_, we made humanized transgenic rescues and compared directly with transgenic expression of nematode *unc‐49B*. Finally, we made specific, novel, epilepsy‐associated point mutations in the *unc‐49* gene and phenotypically analyzed for loss of function. These data indicate that *C. elegans* can be used as a genetic model to screen for epilepsy mutation pathogenicity and specifically identify two GABA_A_ epilepsy mutations as loss of function.

## 2 METHODS

### 2.1 Nematode culture and strains

*Caenorhabditis elegans* were cultured at 20°C on Nematode Growth Media (NGM) agar plates supplemented with 50 μg/mL kanamycin and 100 U/mL nystatin suspension and with OP50 *Escherichia coli* as a food source.^20^ The following strains were used in this study: Bristol N2 (wild‐type), CB407 *unc‐49 (e407)*, CB382 *unc‐49 (e382)*,^18^ FX5487 *unc‐49 (tm5487)*, AMG733 *unc‐49 (ulv30)*, PHX6890 *unc‐49* C167W *(syb6890)*, and PHX6987 *unc‐49* G254D *(syb6987)*.

### 2.2 Generation of point mutations and null mutation of *unc‐49*

The complete *unc‐49* deletion was created by CRISPR‐Cas9 gene editing utilizing two single‐guide RNA (sgRNA) sites designed using the online CRISPR guide RNA selection tool.^22^ The 5′ and 3′ guides were 5′‐acataaaagtctaaatcttt‐3′ and 3′‐gattgagattaatgaaggtc‐5′, respectively, and created a clean deletion of the entire *unc‐49* open reading frame. Gonadal injections of recombinant purified Cas9 and sgRNAs were performed by Magnitude Biosciences (Durham, UK) and were confirmed by PCR and DNA sequencing.

Transgenic worms expressing either *unc‐49B* or human GABA_A_ were made as follows. First, a worm expression vector for *unc‐49* was made utilizing previously generated worm expression Gateway DEST vectors,^23^ replacing *Prab‐3* with *Punc‐49*. For the *unc‐49* promoter, a large ~4.7 kb fragment upstream of the *unc‐49* gene was subcloned using the following primers (5′‐gaagtagtttctgggtccgcc‐3′ and 3′‐cttcttcttcgaggtaagagctctt‐5′), as it has been previously reported that complete transgenic rescue of existing *unc‐49* mutant phenotypes requires substantial 5′ flanking DNA.^18^ Codon‐optimized *unc‐49B* or human GABA_A_ cDNA sequences were made synthetically in pDONR201 entry vectors and subsequently recombined into the DEST vector to produce *Punc‐49* expression vectors. For human GABA_A_, α1 (GABRA1), β3 (GABRB3), and γ2 (GABRG2) subunits were all constructed separately, each under the control of the *Punc‐49* promoter, and injected at equal levels for transgenic expression. The expression vectors were microinjected by SUNY Biotech (Fuzhou, China) into the gonads of the *unc‐49 ulv30* deletion mutant hermaphrodites, along with a *Pmyo‐2*::mCherry reporter to identify transgenic progeny. For each transgenic strain, at least three individual independently derived lines were isolated and phenotypically analyzed.

Finally, individual homozygous point mutations (C167W and G254D) of *unc‐49* were inserted into the genome of our Bristol N2 wild‐type worms by CRISPR‐Cas9 gene editing by SUNY Biotech (Fuzhou, China). For the C167W mutation (*syb6890*), an additional silent mutation was inserted to change an AflIII cut site to HaeIII. For the G254D mutation (*syb6987*), an additional silent mutation was inserted to change an SspI cut site to SpeI. Successful gene editing was confirmed by DNA sequencing.

### 2.3 Phenotypic analysis

Experiments were performed on young adult hermaphrodites in a temperature‐controlled room at 20°C. Phenotypic analysis was performed as previously described for *unc‐49 loss‐of‐function* mutations. Coordinated locomotion was assessed visually, unblinded by counting thrashing or body bends.^7^ Briefly, for thrashing, individual worms were placed in 200 μL Dent's solution (140 mM NaCl, 6 mM KCl, 1 mM CaCl_2_, 1 mM MgCl_2_, 5 mM Hepes, pH 7.4 with 0.1% bovine serum albumin) in a 35‐mm Petri dish and thrashes per minute were calculated after 10 minutes in solution. One thrash was defined as one complete movement from maximum to minimum amplitude and back again. For body bends, individual worms were placed on an unseeded NGM plate and body bends per minute were calculated after 10 min of acclimation. One body bend was defined as one complete sinusoidal movement from maximum to minimum and back again (through the middle axis of the worm). Worm seizure‐like activity was assessed by convulsion assay.^3^ Worms were incubated in M9 buffer (22 mM KH2PO4, 42 mM Na2HPO4, 85.5 mM NaCl, 1 mM MgSO4 with 0.1% bovine serum albumin) supplemented with 7 mg/mL pentylenetetrazol (PTZ) for 20 min. The number of head‐bobbing convulsions per 30 second interval was then recorded. Loss of GABAergic neuron function was assessed by shrinker assay.^24^ In response to a gentle tap on the nematode head animals that failed to move backward, instead displaying body‐wall muscle contraction, were scored as shrinker. For all phenotypic experiments, at least 30 worms per strain were compared from three independent experimental repeats.

### 2.4 Statistical analysis

Significance for thrashing and convulsions were assessed by one‐way analysis of variance (ANOVA) with Tukey post hoc test for multiple comparisons. Significance for shrinker phenotype was assessed by two‐tailed Fisher's exact test.

## 3 RESULTS

**FIGURE 1:**
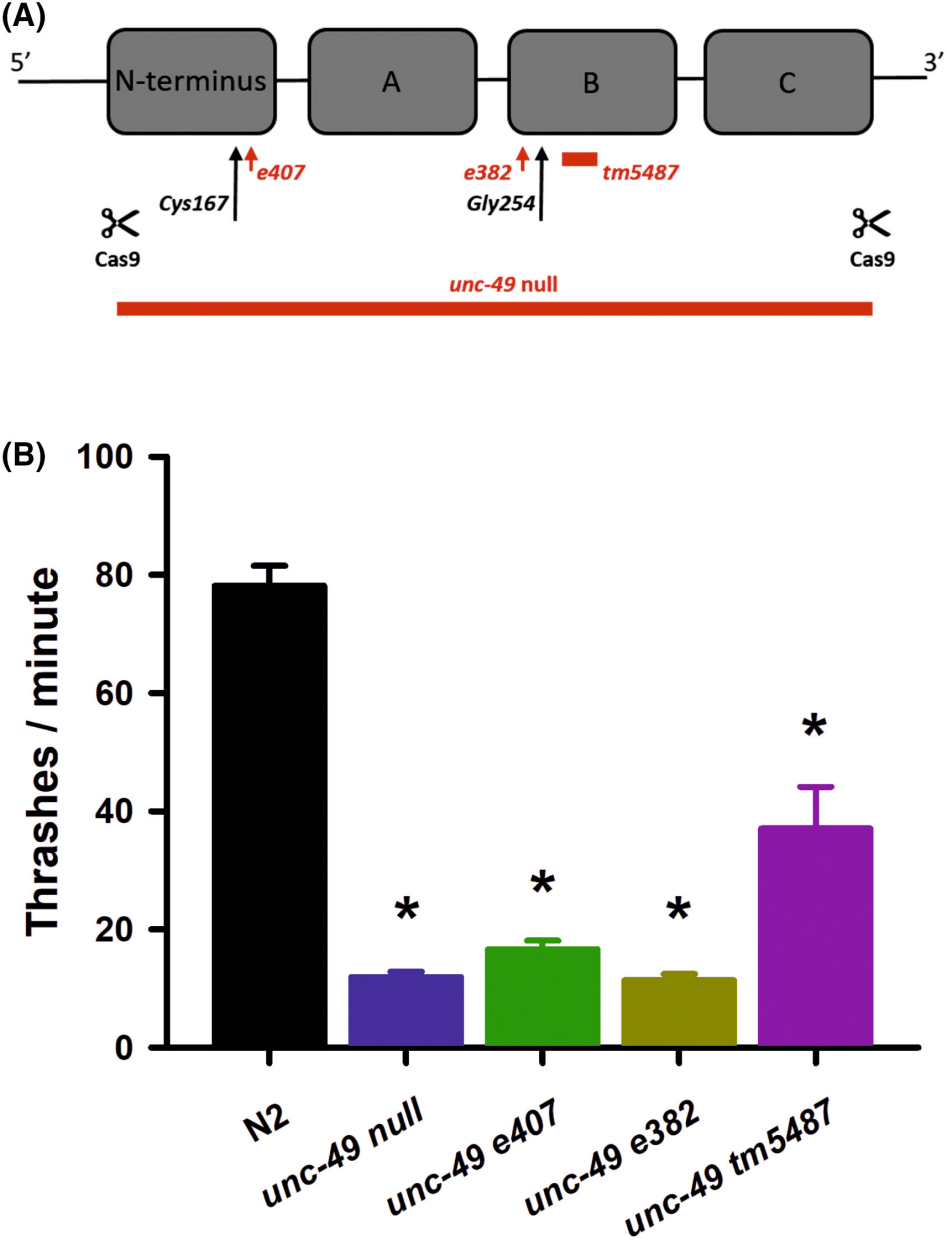
Generation and locomotor analysis of a complete *unc‐49* gene deletion. (A) Cartoon schematic outline of the *unc‐49* gene structure and existing and new mutations used in this study. Three distinct *unc‐49* subunits (UNC‐49A, UNC‐49B, and UNC‐49C) are assembled from a common N‐terminus combined with either the A, B, or C fragment at the C‐terminus. The *e407* allele is a nonsense mutation affecting all three subunits, whereas *e382* (missense) and *tm5487* (small deletion) affect UNC‐49B only. We generated a complete *unc‐49* deletion (*unc‐49 null*) excising all coding fragments and introduced novel transgenic mutations of Cys167 and Gly254 into the endogenous gene. (B) Locomotion was quantified by counting thrashes per minute of worms in solution. In comparison to Bristol N2 wild types, our new *unc‐49 null* mutant as well as the *unc‐49 e407, unc‐49 e382*, and *unc‐49 tm5487* alleles had significantly defective locomotion (indicated by *). None of the *unc‐49* alleles were significantly different from each other. Comparisons made by one‐way ANOVA with Tukey post hoc comparisons (*p* < 0.01, *N* = 30 for each strain). Data shown as mean ± SEM.

The *C. elegans* GABA_A_ receptor homologue *unc‐49* encodes three separate receptor subunits from the same locus.^18^ A large upstream promoter fragment drives expression of discrete individual subunits comprised of a common N‐terminal domain spliced onto three individual downstream components to create UNC‐49A, UNC‐49B, and UNC‐49C proteins (Figure 1A). While expression of UNC‐49A is thought to be negligible, UNC‐49B and UNC‐49C are expressed at high levels where they can form homomeric or heteromeric receptors. A number of mutations associated with the gene have been isolated and characterized. The *e407* allele is a nonsense mutation in the N‐terminal domain common to all three isoforms and, thus, is predicted to produce truncated protein subunits and a loss of function. The *e382* and *tm5487* alleles are missense and small deletion mutations, respectively, specifically in the UNC‐49B subunit. All of these mutations are viable, but can result in reduced, uncoordinated locomotion (*e407, e382*), a shrinker phenotype (*e407, 382*), and hypersensitivity to PTZ‐induced convulsions (*e407*).^3^, ^8^, ^18^, ^20^, ^21^, ^25^, ^26^ Given the complexity of the *unc‐49* gene locus and to investigate GABA_A_ receptors in epilepsy in a clean genetic background, we created an *unc‐49* null allele where the entire coding frame of all three subunits was excised (Figure 1A). Using sgRNAs targeting both ends of the locus for Cas9 excision, we allowed the DNA to repair itself through non‐homologous end‐joining resulting in a complete absence of the entire *unc‐49* reading frame. Gene‐edited *C. elegans* were identified and genotyped by DNA sequencing (Figure S1).

**FIGURE 2:**
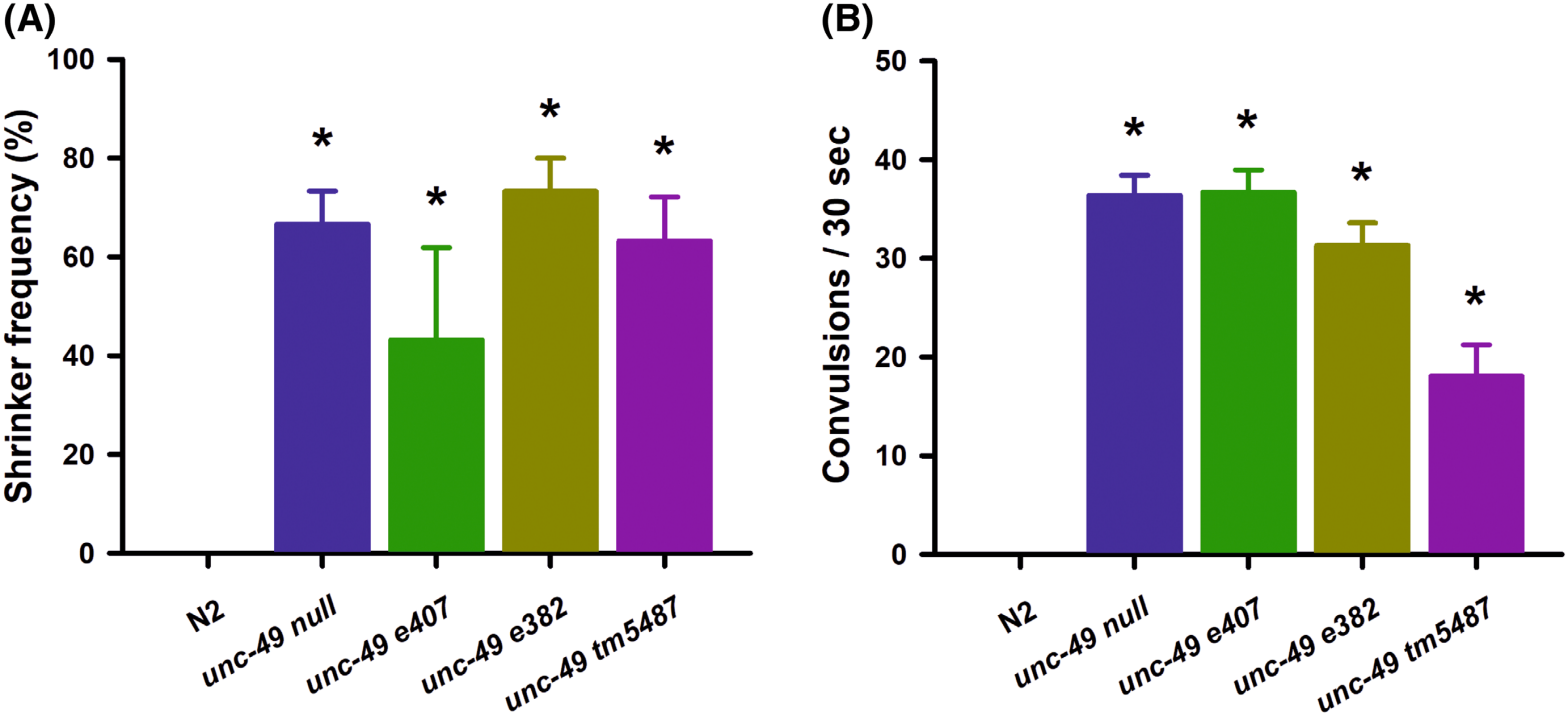
Analysis of shrinker and convulsion frequencies of a complete *unc‐49* deletion. (A) Shrinker frequency was quantified by recording a lack of backward movement to a gentle tap to the animals head. All *unc‐49* mutants had a significant increase (*) in shrinker frequency in comparison with Bristol N2 wild types, but were not different from each other. Comparisons made by Fisher's exact test (*p* < 0.01, *N* = 30 for each strain). Data shown as mean ± SEM. (B) Convulsion frequency was quantified following a 15‐min exposure to 7 mg/mL PTZ by scoring the number of head‐bobbing convulsions over a 30‐s period. All *unc‐49* mutants had a significant increase (*) in convulsion frequency in comparison with Bristol N2 wild types. The *tm5487* allele also showed less convulsions than either the *unc‐49 null* and *e407* alleles. Comparisons made by one‐way ANOVA with Tukey post hoc comparisons (*p* < 0.01, *N* = 30 for each strain). Data shown as mean ± SEM.

Having generated a complete *unc‐49* deletion, we wanted to characterize phenotypically our novel allele against existing *unc‐49* mutations. We first quantified locomotion by thrashing in solution, determining that our *unc‐49* null allele also reduced nematode thrashing, but to an equivalent extent to that seen for existing *unc‐49* mutations (Figure 1B). To verify that the movement deficiency was not specific for thrashing, we also quantified rate of body bends on a solid agar surface, again finding a decrease in locomotion that was indistinguishable from existing mutations (Figure S2). Worms lacking GABA function, including *unc‐49* mutants, are also well characterized as shrinkers.^21^, ^27^ We next quantified the percentage of worms displaying a shrinker phenotype in our *ulv30* null allele, determining that our mutant was similar to that seen with existing *unc‐49* mutations (Figure 2A). Finally, more specifically related to epilepsy, we quantified the number of PTZ‐induced convulsions in our *ulv30* null mutant and found that they were statistically equivalent to the *e407* allele (Figure 2B). We also confirmed an increase in convulsions for the *e382* and *tm5487* alleles, although the *tm5487* allele was slightly less sensitive to PTZ (*p* < 0.01). Therefore, we conclude that our *unc‐49* deletion is a complete loss of function and that the existing mutants, including those only affecting the UNC‐49B subunit are functionally equivalent. This supports the hypothesis that the UNC‐49B subunit is absolutely required for the receptor function, either via a homomeric or heteromeric receptor.^18^, ^19^

**FIGURE 3:**
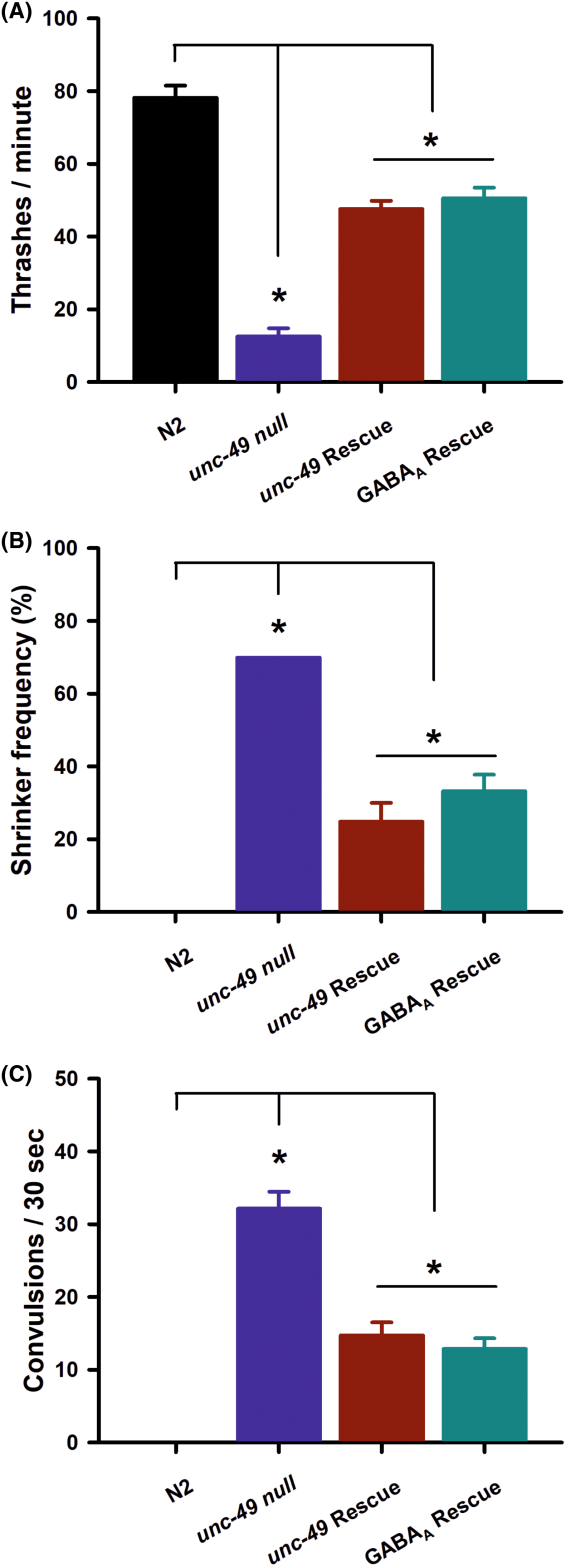
Transgenic expression of *unc‐49B* or the human GABA_A_ receptor partially rescues the *unc‐49 null* phenotypic deficits. (A) Locomotion was quantified by counting thrashes per minute of worms in solution. Transgenic expression of either *unc‐49B* or the human GABA_A_ receptor in the *unc‐49* null background significantly increased locomotion (*); however, locomotion was lower than that seen for wild types. Comparisons made by one‐way ANOVA with Tukey post hoc comparisons (*p* < 0.01, *N* = 30 for each strain). Data shown as mean ± SEM. (B) Shrinker frequency was quantified by recording a lack of backward movement to a gentle tap to the animals head. Transgenic expression of either *unc‐49B* or the human GABA_A_ receptor in the *unc‐49* null background significantly decreased shrinker frequency (*); however, shrinker frequency was greater than that seen for wild types. Comparisons made by Fisher's exact test (*p* < 0.01, *N* = 30 for each strain). Data shown as mean ± SEM. (C) Convulsion frequency was quantified following a 15‐min exposure to 7 mg/mL PTZ by scoring the number of head‐bobbing convulsions over a 30‐s period. Transgenic expression either *unc‐49B* or the human GABA_A_ receptor in the *unc‐49* null background significantly decreased convulsion frequency (*); however, convulsion frequency was greater than that seen for wild types. Comparisons made by one‐way ANOVA (*p* < 0.01, *N* = 30 for each strain). Data shown as mean ± SEM.

We next wanted to determine whether we could create a humanized GABA_A_ receptor strain by transgenically expressing human GABA_A_ subunits (GABRA1, GABRB3, GABRG2) in the *unc‐49 ulv30* null background and quantifying for rescue of phenotypic deficits. We compared the level of rescue in humanized worms directly to transgenic expression of an *unc‐49B* construct in the same *ulv30* null background. In comparison to Bristol N2 wild‐type, the expression of either *unc‐49B* or human GABA_A_ was able to improve the locomotion rate of the *unc‐49 ulv30* nulls (Figure 3A). Neither transgenic strains, however, were able to restore locomotion rates fully to wild‐type levels. This partial phenotypic rescue was evident for all independently derived transgenic lines (Figure S3). Furthermore, analysis of both the shrinker phenotype percentage (Figure 3B) and PTZ‐stimulated convulsions (Figure 3C) showed consistently similar results. Transgenic expression of either *unc‐49B* or human GABA_A_ therefore significantly improved the phenotypes, but not completely to a wild‐type level.

**FIGURE 4:**
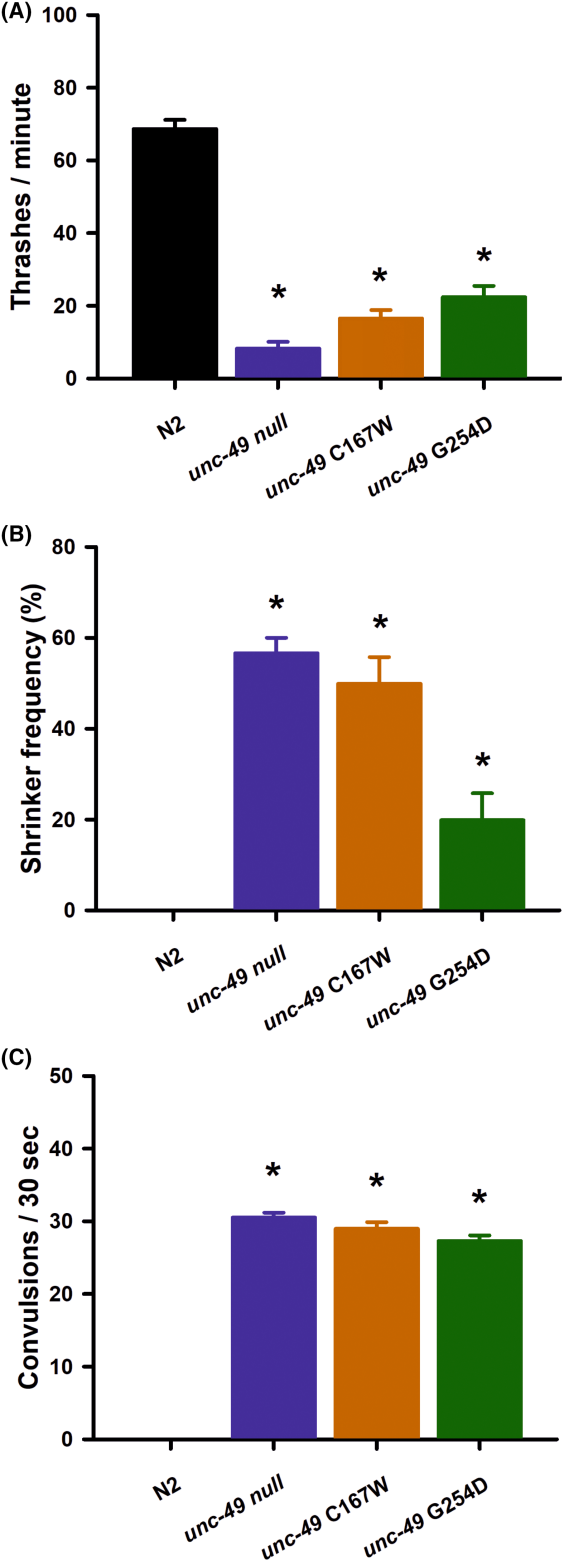
Epilepsy‐linked GABA_A_ receptor point mutations are functionally null. (A) Locomotion was quantified by counting thrashes per minute of worms in solution. Similar to the *unc‐49* nulls, either a C167W or G254D point mutation in the *C. elegans unc‐49* gene (orthologous to human C166W and G251D, respectively) significantly reduced nematode locomotion (*) in comparison with wild‐type worms. Comparisons made by one‐way ANOVA with Tukey post hoc comparisons (*p* < 0.01, *N* = 30 for each strain). Data shown as mean ± SEM. (B) Shrinker frequency was quantified by recording a lack of backward movement to a gentle tap to the animals head. Similar to the *unc‐49* nulls, the C167W or G254D point mutations in *unc‐49* significantly increased shrinker frequency (*) in comparison with wild‐type worms. Comparisons made by Fisher's exact test (*p* < 0.01, *N* = 30 for each strain). Data shown as mean ± SEM. (C) Convulsion frequency was quantified by following a 15‐min exposure to 7 mg/mL PTZ by scoring the number of head‐bobbing convulsions over a 30‐s period. In comparison to wild‐type worms, the C167W or G254D point mutations in *unc‐49* significantly increased shrinker frequency (*) to a level indistinguishable from *unc‐49* null. Comparisons made by one‐way ANOVA (*p* < 0.01 *N* = 30 for each strain). Data shown as mean ± SEM.

Finally, we tested the appropriateness of *C. elegans* as a model for analyzing individual GABA_A_ receptor mutations for functional defects. To that end, we first used ClinVar (https://www.ncbi.nlm.nih.gov/clinvar/) to identify missense mutations in GABRA1 linked with various epileptic phenotypes^28^ and selected two specific single nucleotide variants to investigate further. We chose the G251D mutation as a positive control expected to cause functional defects. Gly251 is contained within the M2 region of the transmembrane domain (Figure S4), and other Gly251 mutations have been determined experimentally to alter GABA receptor currents.^29^ ClinVar gives no further information for G251D; however, the G251S mutation is linked with DEE19 (developmental and epileptic encephalopathy, 19), ECA4 (epilepsy, childhood absence 4), EIG13 (epilepsy, idiopathic generalized, susceptibility to, 13), and IGE (idiopathic generalized epilepsy) (https://www.ncbi.nlm.nih.gov/clinvar/). We also chose the C166W mutation as a single nucleotide variant with unknown pathogenicity and no previous experimental investigations. Similar to the Gly251, ClinVar links this specific mutation with ECA4, EIG13, and IGE (https://www.ncbi.nlm.nih.gov/clinvar/). Both amino acids are conserved residues in all *C. elegans* GABA_A_ receptors and Cys166 is further conserved in all human GABA_A_ receptors (Figure S5). To ensure that there were no potential issues with overexpression, we used CRISPR‐Cas9 editing to make single‐point mutations in the orthologous amino acids in the *unc‐49* gene (C167W and G254D). Both epilepsy‐linked point mutations were found to reduce *C. elegans* locomotion rates to a level similar to the *unc‐49* null (Figure 4A). Interestingly, the G254D mutation exhibited slightly better locomotion rates than the null worms (*p* = 0.03), but was statistically indistinguishable from the C167W mutant. In agreement with this result, both mutations were also found to increase shrinker phenotypes (Figure 4B) and PTZ‐stimulated convulsion frequency to a similar level with the *unc‐49* null (Figure 4C). In summary, both amino acid point mutations appear to be acting as *loss of function* and possibly equivalent to a complete functional deletion of the *unc‐49* gene.

## 4 DISCUSSION

There are currently over 150 annotated epilepsy‐associated variants in the major γ‐aminobutyric acid type A (GABA_A_) receptor subunits^14^, ^15^; however, confirmation of functional defects is lacking for the vast majority of these variants. Various websites are available for predicting pathogenicity for single nucleotide polymorphisms^30^, ^31^, ^32^; however, these remain predictions that can prove conflicting and ideally require in vivo validation. In this paper, we functionally characterize in vivo two specific point mutations in the GABA_A_ receptor that have been linked with epilepsy, concluding that they both are *loss of function* and possibly act as functional null mutations. Generation of point mutations by gene editing directly precludes any potential confounding issues associated with transgenic expression, and thus accurately reflect the impact of the polymorphism.

GABA_A_ receptors are formed as pentameric assemblies of subunits around a central chloride permeable pore.^33^ In mammals, the majority of GABA_A_ receptors are composed of two α subunits, two β subunits, and one γ subunit; however, there are up to 19 distinct subunits that can be assembled resulting in a wide variety of channel isoforms with distinct pharmacological and physiological properties. The Gly251 residue of the α1 subunit (GABRA1) is located within the M2 region of the transmembrane domain of the channel and was selected in this study as a positive control expected to inhibit function. Indeed, a G251S mutation was previously experimentally shown to reduce GABA receptor current by 2.6‐fold in *Xenopus* oocytes.^29^ We predicted that mutation of Gly251 to an acidic amino acid such as aspartate (G251D) would also interfere with chloride ion flux through the channel, either by pore occlusion or via electrical repulsion. In agreement with this prediction, our results indicate that the orthologous *C. elegans* G254D mutation was mostly indistinguishable from the complete deletion of the *C. elegans unc‐49* gene. In the worm, the in vivo receptor is an UNC‐49B/C heteromer^19^ and the G254D mutation only exists in the UNC‐49B subunit. Therefore, the unaffected UNC‐49C subunit here may account for the potential residual channel activity, as there were statistical differences evident between the *unc‐49* null and the G254D mutants.

In contrast to Gly251, the Cys166 residue is located in the extracellular domain of the α1 subunit (GABRA1), as a component of the cys‐loop of the receptor. Given the location of the amino acid, predictions on this mutations potential pathogenicity have been more variable. Indeed, ClinVar predicts uncertain clinical significance for C166W, whereas SIFT and PolyPhen predict the mutation is probably damaging. Importantly, there are no in vivo investigations into the mutation to verify predictions one way or the other. Our results indicate that the orthologous *C. elegans* C167W mutation was also functionally equivalent to the complete gene deletion, possibly demonstrating a complete *loss of function*. From our phenotypic data, however, it remains unclear whether this *loss of function* derives from altered receptor activation or ion flux. Interestingly here, the mutation is a component of the conserved N‐terminal domain of the *unc‐49* gene locus, meaning that the mutation would exist in all subunits of the UNC‐49B/C heteromer. In humans, however, the GABRA1 mutation would only exist in the α1 subunit of the GABA_A_ receptor, likely indicating that some residual channel activity may be retained in epilepsy patients with this mutation.

In *C. elegans*, seven identified genes show orthology to GABA‐activated cys‐loop channels^16^, ^34^; however, there is little known about their roles in epileptic phenotypes. Two of these (*exp‐1* and *lgc‐35*) encode excitatory cationic channels,^35^, ^36^ whereas four (*gab‐1*, *lgc‐36*, *lgc‐37*, and *lgc‐38*) are predicted GABA_A_ receptor subunits linked with olfactory adaptation, responses to nicotine and resistance to a novel anticonvulsant, chlorothymol.^10^, ^37^, ^38^ In contrast to these other six genes, a substantial amount is known about the GABA_A_ receptor *unc‐49*. The *unc‐49* receptor is assembled mainly from homomeric and heteromeric assemblies of the UNC‐49B and UNC‐49C subunits, while the contribution of the UNC‐49A subunit appears to be negligible. Most existing mutations for *unc‐49* either affect all subunits or *unc‐49B* specifically; however, there are no true deletions for *unc‐49*. In this paper we have created and characterized functionally a novel full deletion mutant of the GABA_A_ receptor gene *unc‐49* in *C. elegans* by CRISPR‐Cas9 gene editing. The complete removal of the entire coding region, including all exons of the N‐terminus and each distinct C‐terminal subunits thus excluded any complications arising from possible residual domain expression. In comparison with previously identified *unc‐49 loss‐of‐function* mutations for a reduction in coordinated locomotion, an increase in shrinker frequency, and an increase in PTZ‐induced convulsions, we found that our full deletion was no stronger than previous mutations that either affected all three isoforms or only the UNC‐49B subunit. This indicates that these previous mutations are functionally null and support the hypothesis that the UNC‐49B subunit is absolutely critical for receptor function. Additionally, our novel *unc‐49* null allele represents a clean genetic model for further functional investigations in vivo.

In contrast to those data, rescue of the complete *unc‐49* deletion with a transgene expressing *unc‐49B* only showed partial rescue for all phenotypes investigated, for which there may be a number of possible explanations. This may be an explicit consequence of the modulatory requirement of UNC‐49C, and/or UNC‐49A, for full receptor function in vivo. While UNC‐49B alone is sufficient to form a functional receptor both in vitro and in vivo, UNC‐49C plays an important regulatory role.^19^ Alternatively, it remains possible that subtle differences in expression level or promoter activity may be requisite for physiological channel function. Support for this interpretation comes from the additional evidence that humanized *unc‐49* nulls expressing the α1 (GABRA1), β3 (GABRB3), and γ2 (GABRG2) subunits were phenotypically indistinguishable from the transgenic rescues with *unc‐49B*. More precise regulation of subunit expression may be required for a full phenotypic rescue in humanized worms. Given that GABRA1 has only 36% sequence identity with the individual *unc‐49* subunits, it also remains possible that *unc‐49* cannot be completely replaced by the human GABA_A_ receptor. Further experiments would be required to elucidate these issues fully.

Epilepsy is a heterogeneous group of disorders that cause various neurological deficits such as seizures.^1^, ^2^ The root causes of epilepsy are varied, but include genetic mutation. Advances in genomic sequencing have increased exponentially the identified genes and mutations that may contribute to epileptic phenotypes. This increase has concomitantly resulted in a dearth of pathogenicity confirmation of individual mutations, presumably because of the experimental time and expense involved. Various genetic models for epilepsy have evolved including *Drosophila*, zebrafish, and *C. elegans*.^3^, ^4^, ^5^, ^6^ Our data support the use of *C. elegans* as a valuable epilepsy model to test pathogenicity of individual amino acids and confirm loss of functionality for two individual mutations. In addition, we have generated and characterized a humanized GABA_A_ receptor worm model that can partially replace *unc‐49* function and this model could be used as an alternative platform for mutational analysis and/or pharmacological testing in vivo at a whole organism level.

## CONFLICT OF INTEREST STATEMENT

None of the authors has any conflict of interest to disclose. We confirm that we have read the Journal's position on issues involved in ethical publication and affirm that this report is consistent with those guidelines.

## Supporting information


Appendix S1.

## Data Availability

The data that support the findings of this study are available from the corresponding author upon reasonable request.
