# Supplementary material for: Functional analysis of epilepsy‐associated GABAA receptor mutations using Caenorhabditis elegans
Source: Epilepsia Open. 2024 May 30;9(4):1458–66. doi: 10.1002/epi4.12982 (PMC11296113; doi:10.1002/epi4.12982)
Supplement: Supplementary file 1 — Appendix S1. [file EPI4-9-1458-s001.pdf]

Gadhia *et al.* Supplemental information

Figure S1: Sequence of *unc-49 ulv30* null strain.

A complete genetic deletion of the *unc-49* locus was created by CRISPR-Cas9 gene editing. The figure shows the genome sequence of wild-type and *unc-49 ulv30* null worms. Flanking regions to the mutation are highlighted in yellow. Altered sequence is in red.

Figure S2: Locomotor analysis of a complete *unc-49* deletion.

Locomotion was quantified by counting body bends per minute of worms on solid agar. In comparison to Bristol N2 wild-types, our new *unc-49 null* mutant as well as the *unc-49 e407, unc-49 e382* and *unc-49 tm5487* had significantly defective locomotion (indicated by *). None of the *unc-49* alleles were different from each other. Comparisons made by one-way ANOVA with Tukey post-hoc comparisons ($P<0.01$, N=30 for each strain). Data shown as mean ± SEM.

Figure S3: Transgenic expression of *unc-49B* or human $GABA_A$ receptors in the *unc-49 ulv30* null background.

Individual transgenic lines were generated where *unc-49B* or human $GABA_A$ receptors were expressed under the control of the endogenous *unc-49* promoter in the *unc-49 ulv30* null background. Three individual lines were isolated for *unc-49B* and four for human $GABA_A$ receptors. Comparisons were made between transgenic lines for **(A)** locomotion quantified by thrashing per minute, **(B)** shrinker frequency and **(C)** convulsion frequency in response to PTZ exposure. All lines were statistically equivalent to each other. Comparisons were made by one-way ANOVA with Tukey post-hoc comparisons (thrashing, convulsions) or by Fisher's exact test (N=30 for each individual line). Data shown as mean ± SEM.

Figure S4: Location of epilepsy-associated mutations in the $GABA_A$ receptor structure

Structure of the assembled human $GABA_A$ receptor was derived from the PDB file 6T6D [1]. The two α subunits are indicated in green, the two β subunits are in blue and the single γ subunit is in purple. Individual epilepsy-associated mutations investigated in this paper (α subunit mutations Cys166 and Gly251) are represented by red spheres.

Figure S5: $GABA_A$ receptor sequence alignments

Sequence alignments of 3 human α (GABRA1), β (GABRB3) and γ (GABRG2) $GABA_A$ receptor subunits along with 3 *C. elegans unc-49* isoforms (UNC-49A, UNC-49B and UNC-49C). Sequence identity between GBRA1 and UNC-49A, UNC-49B and UNC-49C were all 36%. The individual epilepsy-associated mutations investigated in this paper (Cys166 in GABRA1, Cys167 in *unc-49*; Gly251 in GABRA1, Gly254 in *unc-49*) are highlighted in yellow.

Supplemental References

1. Zhu S, Noviello CM, Teng J, Walsh RM, Kim JJ and Hibbs RE. Structure of a human synaptic $GABA_A$ receptor. Nature 2018;559:67-72.

Figure S1

>wild-type
TGTAGAGCGAGGCCTGCCTGCCTACCGGCCGCCTATATAGTTCTATTTGGTCTGAGCAA

GTAATGGTAGCCACGCGGGTAGGTATAATTCAGACGATTCTGTGCCTACATAAAAGTCT

AAATCTTTTAATCCGCAAACTTTAAGAAGGTAGTTATTTTGCTGCTAATTGTTAAGCCTA

AGTCTAGGTCTAAGCCTAAAAC **… +11,306bp …**

TTCATCTTGTTCAACACTCTCTTCTGGCTGATTCTACTGTACAAATCCAAGCGTCTGCCG

TATATTAGTGAACACGAGGGTGACCGTTGCGATGCTCCAGACCTTCATTAATCTCAATCC

AACTTCCTCATCATTTTCCATTTCGAATATCTCTTTTTCTTGCACAGAAGCCTTTTTTCGT

TTTTTTTTATTGATTTATT

>*unc-49 ulv30* null
TGTAGAGCGAGGCCTGCCTGCCTACCGGCCGCCTATATAGTTCTATTTGGTCTGAGCAA

GTAATGGTAGCCACGCGGGTAGGTATAATTCAGACGATTCTTCATTAATCTCAAAATCTC

AATCTCAATCCAATCTCAATCCAACTTCCTCAATAAAATAAAATCTCAATACCTTCATTAA

TCTCAATCCAACTTCCTCATCATTTTCCATTTCGAATATCTCTTTTTCTTGCACAGAAGCC

TTTTTTCGTTTTTTTTTATTGATTTATT

Figure S2

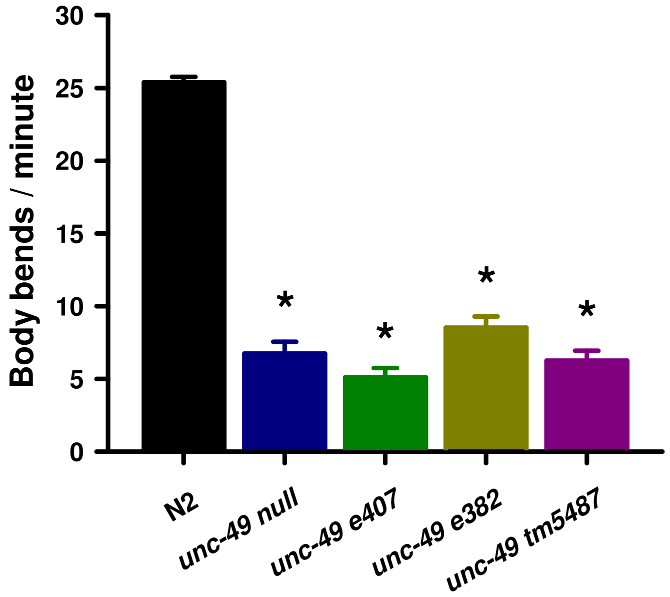

Body bends / minute
30
25
20
15
10
5
0
*
*
*
*
N2
unc-49 null
unc-49 e407
unc-49 e382
unc-49 tm5487

Figure S3

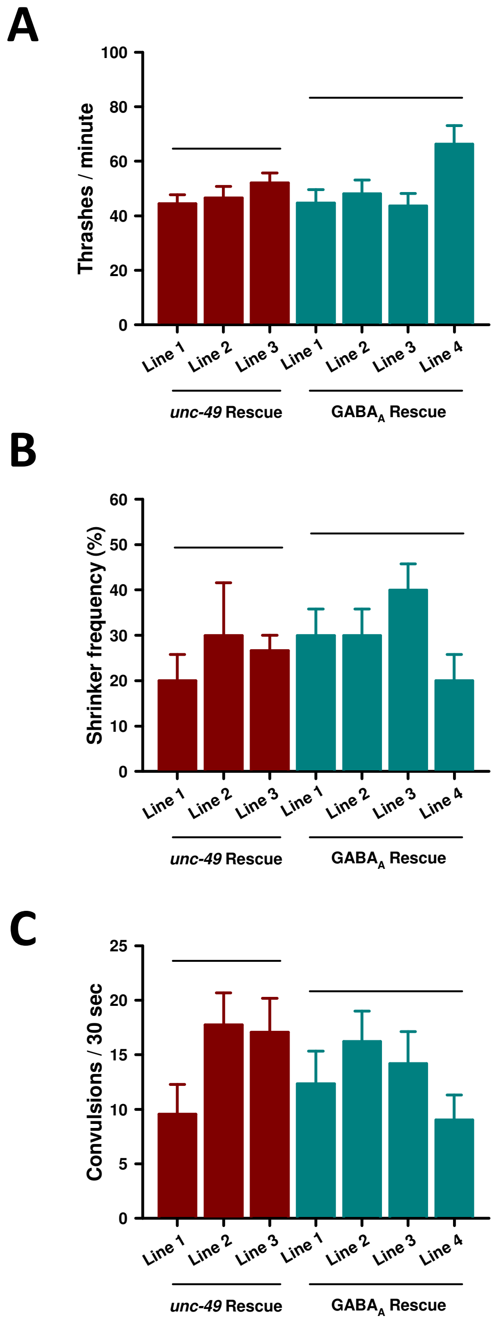
A
Thrashes / minute
100
80
60
40
20
0
Line 1
Line 2
Line 3
Line 1
Line 2
Line 3
Line 4
unc-49 Rescue
GABA$_A$ Rescue
B
Shrinker frequency (%)
60
50
40
30
20
10
0
Line 1
Line 2
Line 3
Line 1
Line 2
Line 3
Line 4
unc-49 Rescue
GABA$_A$ Rescue
C
Convulsions / 30 sec
25
20
15
10
5
0
Line 1
Line 2
Line 3
Line 1
Line 2
Line 3
Line 4
unc-49 Rescue
GABA$_A$ Rescue

Figure S4

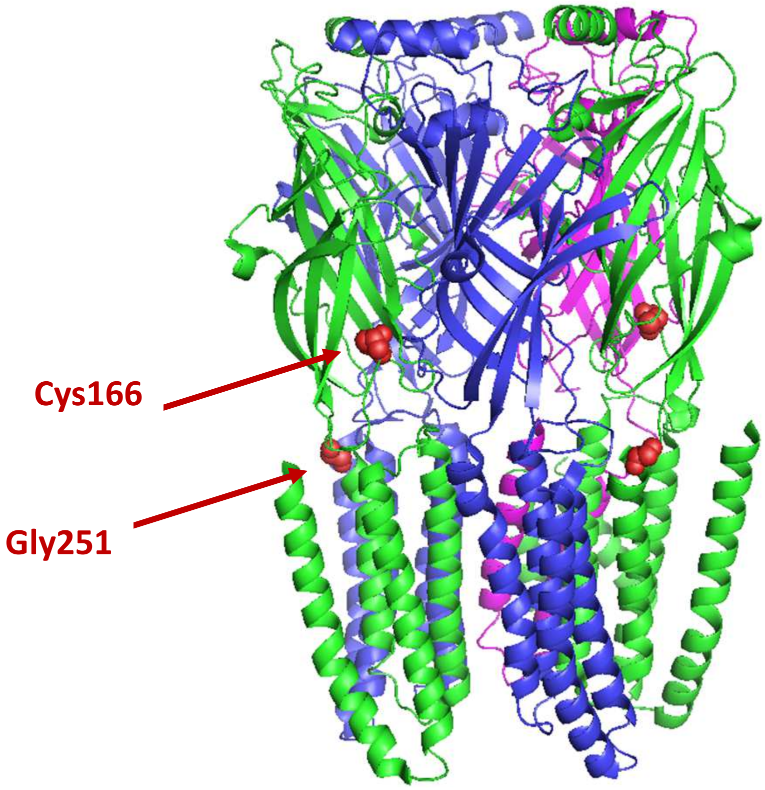

Figure S5

```
GBRA1    ------------MRKSPGLSDCLWAWILLLSTLTGRSYGQPS---LQDELKDNTTVFTR---------ILDRLLDG--YDNRLRPGLGERVTEVKTDIFVTSFGPVSDH 83
GBRB3    ---------------MWGLAGGRLFGIFSAPVLVAVVCCAQS-----VNDPGNMSFVKE---------TVDKLLKG--YDIRLRPDFGGPPVCVGMNIDIASIDMVSEV 78
GBRG2    MSSPNIWSTGSSVYSTPVFSQKMTVWILLLLSLYPGFTSQKSDDDYEDYASNKTWVLTPKVPEGDVTVILNNLLEG--YDNKLRPDIGVKPTLIHTDMYVNSIGPVNAI 107
UNC-49A  ------------------MARPFTLIVLLSAHLCLHVVVTQD-----EDSHINTQLLSS---------VLDRLTNRTTYDKRLRPRYGEKPVDVGITIHVSSISAVSEV 77
UNC-49B  ------------------MARPFTLIVLLSAHLCLHVVVTQD-----EDSHINTQLLSS---------VLDRLTNRTTYDKRLRPRYGEKPVDVGITIHVSSISAVSEV 77
UNC-49C  ------------------MARPFTLIVLLSAHLCLHVVVTQD-----EDSHINTQLLSS---------VLDRLTNRTTYDKRLRPRYGEKPVDVGITIHVSSISAVSEV 77
                           ::      ::    *         .          :  ...          ::.* .   ** :***  *   . :   : : *:. *.

GBRA1    DMEYTIDVFFRQSWKDERLKFKGPMTVLRLNNL-------MASKIWTPDTFFHNGKKSVAHNMTMPNKLLRITEDGTLLYTMRLTVRAECPMHLEDFPMDAHACPLKFG 185
GBRB3    NMDYTLTMYFQQYWRDKRLAYSGIPLNLTLDNR-------VADQLWVPDTYFLNDKKSFVHGVTVKNRMIRLHPDGTVLYGLRITTTAACMMDLRRYPLDEQNCTLEIE 180
GBRG2    NMEYTIDIFFAQTWYDRRLKFNSTIKVLRLNSN-------MVGKIWIPDTFFRNSKKADAHWITTPNRMLRIWNDGRVLYTLRLTIDAECQLQLHNFPMDEHSCPLEFS 209
UNC-49A  DMDFTLDFYMRQTWQDPRLAFGSLDLGLSKEIDSLTVGVDYLDRLWKPDTFFPNEKKSFFHLATTHNSFLRIEGDGTVYTSQRLTVTATCPMDLKLFPMDSQHCKLEIE 186
UNC-49B  DMDFTLDFYMRQTWQDPRLAFGSLDLGLSKEIDSLTVGVDYLDRLWKPDTFFPNEKKSFFHLATTHNSFLRIEGDGTVYTSQRLTVTATCPMDLKLFPMDSQHCKLEIE 186
UNC-49C  DMDFTLDFYMRQTWQDPRLAFGSLDLGLSKEIDSLTVGVDYLDRLWKPDTFFPNEKKSFFHLATTHNSFLRIEGDGTVYTSQRLTVTATCPMDLKLFPMDSQHCKLEIE 186
         :*::*: .:: * * * ** : .     *  :             .::* ***:* * **:  *  *  * ::*:  ** :    *:*  * * :.*. :*:* : * *::

GBRA1    SYAYTRAEVVYEWTREP--ARSVVVAEDGS-RLNQYDLLGQTVDSGIVQSSTGEYVVMTTHFHLKRKIGYFVIQTYLPCIMTVILSQVSFWLNRESVPARTVFGVTTVL 291
GBRB3    SYGYTTDDIEFYWRGGD-----KAVTGVERIELPQFSIVEHRLVSRNVVFATGAYPRLSLSFRLKRNIGYFILQTYMPSILITILSWVSFWINYDASAARVALGITTVL 284
GBRG2    SYGYPREEIVYQWKRS-----SVEVGDTRSWRLYQFSFVGLRNTTEVVKTTSGDYVVMSVYFDLSRRMGYFTIQTYIPCTLIVVLSWVSFWINKDAVPARTSLGITTVL 313
UNC-49A  SYGYSILDIMYVSHEKK--SVSTESYELPQFVLQSIKVVNHTQK-----LSSGEYSRLCWFFLFKRNIGFYIIQIYLPSVLIVVISWVSFWLSRDATPARVALGVTTVL 288
UNC-49B  SYGYETKDIDYYWGKKR-TDLEITAVKFDTFQLPQFQPTLYFVNTTKAETSSGKYVRLALEVILVRNMGFYTMNIVIPSILIVTISWVSFWLNREASPARVGLGVTTVL 294
UNC-49C  SYAYSTAEIEYKWCTSKEPNCSTAVKADANIELSSYKFTKICQKRTLASTSSGTYSRLRVSFIFDRDSGFYFLQIFFPASLVVVLSWISFWINRDSAPSRTLIGTMTVL 295
         **.*   :: :                  * . .              ::* *  :   . : *  *:: ::  :*. : . :* :***:. :: .:*. :*  ***

GBRA1    TMTTLSISARNSLPKVAYATAMDWFIAVCYAFVFSALIEFATVNYFTKRGYAWDGKSVV---------------PEKPKKVKDPLIKKNNTYAPTATSYTPNLARGDP- 384
GBRB3    TMTTINTHLRETLPKIPYVKAIDMYLMGCFVFVFLALLEYAFVNYIFFGRGPQRQKKLAEKTAKAKNDRS----KSESNRVDAHGNILLTSLEVHNEMNEVSGGIGDTR 389
GBRG2    TMTTLSTIARKSLPKVSYVTAMDLFVSVCFIFVFSALVEYGTLHYFVSNRKPSKDK-------------------DKKKKNPAPTIDIRPRSATIQMNNATHLQERDEE 403
UNC-49A  TMTTLMTMTNSSMPKVSYVKSIDIFLGVCFMMVFCSLLEYAAVGYISKRMKLVRARKESRMLTPLPHLESLPPKRTLSVPSYFNNTTYRPFYSSTDQTSNLYIP--ESQ 395
UNC-49B  TMTTLITTTNNSMPKVSYVKGLDVFLNFCFVMVFASLLEYAIVSYMNKRLVLRREKRR----------------KAAEQQQRNEMPMFNASPKAANNNADLYFAGHNSS 387
UNC-49C  TETHLMTGTNRRLPPVAYVKAVDVFLGFCYLLVILALIEYACVAYSKKKNEDRRRREK--------------------------KTEHKPAPPTPDILHDVRLA--ECT 376
         * * :    .  :* :.*...:* ::  *: :*: :*:*:. : *          :                                                  :

GBRA1    -GLATIAKS--ATIEPKEVKPETKPPE---------------------PKKTFNSVSKIDRLSRIAFPLLFGIFNLVYWATYLNREPQLKAPTPHQ*---------- 457
GBRB3    NSAISFDNSGIQYRKQSMPREGHGRFLGDRSLPHKKTHLRRRSSQLKIKIPDLTDVNAIDRWSRIVFPFTFSLFNLVYWLYYVN*---------------------- 474
GBRG2    YGYECLDGKDCASFFCCFEDCRTGAWR---------------------HGRIHIRIAKMDSYARIFFPTAFCLFNLVYWVSYLYL*-------------------- 468
UNC-49A  RTTIFSNEDAVPNELTPMLGRSNSQAS---VFLYQTAVISDDEF---GRFWRWLRPSNIDKYSRSLFPSIFVLFNVGYWAYFIRQSQ----IQEEQRN---SQIL*- 488
UNC-49B  MNPLMEIPENCDCRTIPMMQHPRLVTDGAHTLWPAPFARPKKAS---KTCCQRWTPAKIDKLSRYGFPLSFSIFNIVYWLYMKYLSLN---SSDKIQENDKWQQIH* 488
UNC-49C  CN-------AAPTSIIAVIKQSN-------------------------RFC--VSHSHIDIVSRAAFPLVFILFNTLFWLILLYKSKRLPYISEHEGDRCDAPDLH* 449
                                                                :*  :*  **  * :**  :*
```
